# Ovarian Carcinoma: Signal Pathways and Clinical Studies

**DOI:** 10.1002/mco2.70975

**Published:** 2026-11-03

**Authors:** Yanru Long, Houhui Shi, Jiatian Ye, Xiaorong Qi

**Affiliations:** ^1^ Department of Gynecology and Obstetrics Key Laboratory of Birth Defects and Related Diseases of Women and Children (Sichuan University) Ministry of Education West China Second Hospital Sichuan University Chengdu People's Republic of China

**Keywords:** angiogenesis, clinical studies, DNA damage repair pathway, HER2 signaling pathway, notch signaling pathway, ovarian carcinoma, PI3K/AKT/mTOR

## Abstract

Ovarian carcinoma is the most lethal gynecologic malignancy, with a 5‐year survival rate for advanced patients that has remained stubbornly low due to high recurrence and acquired drug resistance. Despite advances in surgery and chemotherapy, a critical gap remains in translating an understanding of the tumor's complex molecular heterogeneity into effective, durable treatments. This review systematically dissects the latest advances in key signaling pathways driving ovarian carcinoma pathogenesis, including the Notch, HER2, DNA damage repair (DDR), PI3K/AKT/mTOR, and angiogenesis (VEGF) pathways, and critically evaluates the clinical progress of corresponding targeted therapies. We particularly focus on the regulatory networks within the tumor microenvironment and the mechanisms of crosstalk between pathways that contribute to treatment resistance. The review synthesizes clinical study outcomes for novel agents such as PARP inhibitors (olaparib and niraparib), antibody‐drug conjugates (e.g., T‐DXd), and anti‐angiogenic therapies (bevacizumab), while analyzing the challenges of primary and acquired resistance. By bridging fundamental pathway biology with emerging clinical data, this review provides a strategic framework for advancing precision medicine and developing rational combination therapies to improve outcomes for patients with this devastating disease.

## Graphical abstract

This review synthesizes current knowledge on the pathophysiological mechanisms driven by the Notch, HER2, DNA damage repair (DDR), PI3K/AKT/mTOR, and angiogenesis (VEGF) pathways in ovarian cancer. A key focus is placed on summarizing subsequent targeted therapeutic strategies, detailing the landscape of associated clinical trials and their developmental phases.

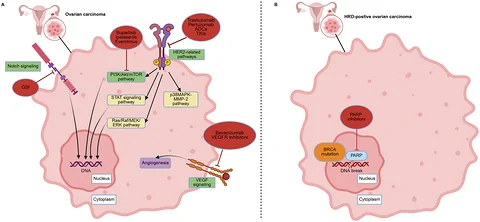

## 1 Introduction

Ovarian carcinoma is one of the most common malignant tumors of the female reproductive system. According to global carcinoma statistics, more than 310,000 new cases of ovarian carcinoma are diagnosed each year, with over 200,000 deaths attributed to the disease during the same period [1, 2]. Updated epidemiological analyses confirm this high burden, with incidence and mortality rates ranking among the highest of female reproductive system malignancies [3, 4]. Ovarian carcinoma incidence shows a distinct age distribution pattern, with the highest incidence occurring in women aged 50–79, primarily postmenopausal women. However, certain specific types, such as germ cell tumors, can also occur in young women and even children [5]. Epidemiological studies have identified several risk factors, including nulliparity, late childbearing, early menarche or late menopause, breast cancer susceptibility gene (BRCA) mutations, Lynch syndrome, endometriosis, obesity, and so on [6, 7, 8, 9, 10].

Ovarian carcinoma is a highly heterogeneous group of diseases with complex pathogenesis. It can originate in the ovaries themselves or in ovarian‐related tissues such as the fallopian tubes and peritoneum. Different subtypes exhibit significant differences in molecular characteristics, clinical behavior, and treatment response [11]. From a histological classification perspective, ovarian carcinoma primarily includes epithelial ovarian carcinoma, germ cell tumors, and sex cord‐stromal tumors, with epithelial ovarian carcinoma accounting for approximately 90% of cases. Epithelial ovarian carcinoma can be further subdivided into subtypes such as high‐grade serous carcinoma (HGSC), endometrioid carcinoma, clear cell carcinoma, and mucinous carcinoma. Among these, HGSC accounts for approximately 70%–80%, endometrioid carcinoma accounts for approximately 10%, clear cell carcinoma accounts for approximately 10%, and mucinous carcinoma accounts for approximately 3%–5% [12, 13]. The classification system for epithelial ovarian carcinoma has gradually improved in recent years, with the most influential being the binary classification model based on molecular characteristics, clinical behavior, and pathological features. This classification system divides epithelial ovarian carcinoma into two major categories, namely, Type I and Type II [14]. Type I ovarian carcinoma typically grows slowly and is often a low‐grade tumor, including low‐grade serous carcinoma, endometrioid carcinoma, clear cell carcinoma, and mucinous carcinoma. This type of ovarian carcinoma often develops from benign or borderline tumors and is associated with specific driver gene mutations, such as KRAS, PTEN, and ARID1A. It has been confirmed that the clinical prognosis for this type of carcinoma is relatively good [15]. Type II ovarian carcinoma, on the other hand, is highly invasive and predominantly consists of high‐grade tumors, primarily HGSC. It typically arises from direct malignant transformation of the epithelial cells at the distal end of the fallopian tubes or the ovarian surface. It is often associated with TP53 mutations and high genomic instability. At the time of diagnosis, it is frequently at an advanced stage, progresses rapidly, and has a poor prognosis [16]. This classification model is more helpful in understanding the pathogenesis of ovarian carcinoma.

Ovarian carcinoma has insidious onset. Clinically, most early cases present with non‐specific symptoms such as abdominal distension and indigestion, which are easily overlooked. Coupled with the lack of effective screening methods, approximately 70% of patients are diagnosed at Stage III or IV [17, 18]. It can thus be concluded that ovarian carcinoma has a poor prognosis, with its incidence and mortality rates ranking among the highest among female reproductive system malignancies. This grim clinical reality makes ovarian carcinoma a major threat to women's health worldwide [3, 4, 19]. Therefore, an increasing number of studies are focusing on exploring treatments for ovarian carcinoma. When treating ovarian carcinoma, it is necessary to comprehensively consider its pathological type, molecular characteristics, patient age and fertility needs, tumor burden, and metastasis status. Currently, the standard treatment for ovarian carcinoma in clinical practice remains primarily based on cytoreductive surgery combined with platinum‐based chemotherapy [20]. Although the initial treatment response rate is high, approximately 70% of patients will eventually experience recurrence and develop platinum‐resistant disease. The prognosis for these patients is extremely poor, with a median progression‐free survival (PFS) of 3–4 months and a median survival of no more than 12 months [21, 22]. More seriously, second‐line treatment options for platinum‐resistant ovarian carcinoma are extremely limited and may cause severe side effects. This highlights the urgency of developing new treatment strategies.

Over the past few decades, a large number of preclinical studies and clinical trials have been conducted to screen potential drug candidates, including monotherapy or combination therapy regimens, or to optimize existing chemotherapy regimens for ovarian carcinoma. Although some progress has been made, breakthroughs in the field of anti‐cancer drug research and development remain insufficient and require further exploration. In recent years, a number of basic studies have shown that there are abnormalities in the regulation of multiple signaling pathway components in ovarian carcinoma. These abnormally activated signaling pathways play a key role in tumor proliferation, metastasis, and the development of drug resistance. Therefore, in‐depth analysis of these dysregulated pathways involved in the development of ovarian carcinoma may provide important evidence for the development of new treatment strategies for ovarian carcinoma. It is encouraging that some innovative drugs based on key signaling pathways in ovarian carcinoma, such as poly (ADP‐ribose) polymerase (PARP) inhibitors and immune checkpoint inhibitors, have shown promising efficacy in clinical trials.

This review systematically describes the basic characteristics of important signaling pathways in ovarian carcinoma, such as the Notch signaling pathway, human epidermal growth factor receptor 2 (HER2)‐related pathways, and the phosphoinositide 3‐kinase/protein kinase B/mammalian target of rapamycin (PI3K/AKT/mTOR) signaling pathway, as well as their role in reshaping the tumor microenvironment (TME). Additionally, this paper also thoroughly analyses the specific molecular mechanisms by which these pathways promote the progression of ovarian carcinoma through the regulation of cell cycle, apoptosis resistance, epithelial‐mesenchymal transition (EMT), and angiogenesis. In terms of treatment strategies, this paper focuses on the progress of targeted drug development based on signal pathways, including the clinical application effects and resistance mechanisms of targeted drugs such as small molecule inhibitors, monoclonal antibodies, antibody‐drug conjugates (ADCs), and PARP inhibitors. Besides, this review also discusses the research progress of combined use of targeted therapy with immunotherapy such as PD‐1/PD‐L1 inhibitors, providing a basis for clinical treatment strategies for ovarian carcinoma. Additionally, this paper summarizes some innovative therapies currently in preclinical research and clinical trial stages, providing a theoretical basis and translational research directions for the development of more effective and precise ovarian carcinoma treatment regimens.

## 2 Key Signaling Pathways in Ovarian Carcinoma, Their Regulatory Mechanisms, and Therapeutic Targeting

This section systematically examines the major signaling pathways that are dysregulated in ovarian carcinoma. For each pathway, we first describe its basic biology and oncogenic role, then detail its specific contribution to tumor progression, metastasis, and immune modulation, and finally review the clinical strategies developed to target it.

### 2.1 Notch Signaling Pathway

#### 2.1.1 Basic Characteristics of the Notch Signaling Pathway

The Notch signaling pathway, as a highly conserved intercellular communication system, plays a crucial role in various physiological and pathological processes. The core components of this pathway include four Notch receptors (Notch1, 2, 3, and 4), five ligands (Jagged1, Jagged2, Dll1, Dll3, and Dll4), and downstream effector molecules in mammals [23]. Notch receptors are synthesized in the endoplasmic reticulum in the form of inactive single peptide precursors, which are then catalyzed by a furin‐like trans‐Golgi network enzyme to undergo the first proteolytic cleavage (S1), forming heterodimers composed of N‐terminal ligand‐accessible Notch extracellular subunit (NEC) and C‐terminal Notch transmembrane fragment (NTM) [24]. Activation of the Notch signaling pathway begins with the binding of a ligand to its receptor. This interaction induces a conformational change, followed by a second cleavage (S2) by a disintegrin and metalloproteases (ADAM) family (such as ADAM10 or ADAM17), releasing the extracellular subunit and generating a membrane‐associated Notch extracellular truncated (NEXT) intermediate [25]. Next, the γ‐secretase complex (dependent on senescent protein) mediates the third cleavage (S3), releasing the intracellular domain of the Notch receptor (NICD). After translocating into the nucleus, NICD binds to the transcription factor CSL (also known as CBF1/Suppressor of Hairless/LAG‐1). This binding converts the co‐repressor complex into a transcriptional activator complex [23]. The complex contains NICD, CSL, mastermind‐like protein (MAML), Ski‐interacting protein (SKIP), and p300, which activate the transcription of downstream target genes such as Hairy Enhancer of Split (HES) family proteins, HES‐related proteins (HEY), cyclin D1/D3, and HER2, thereby regulating basic life processes such as cell fate determination, proliferation, differentiation, and survival [24, 26, 27] (Figure 1).

**FIGURE 1:**
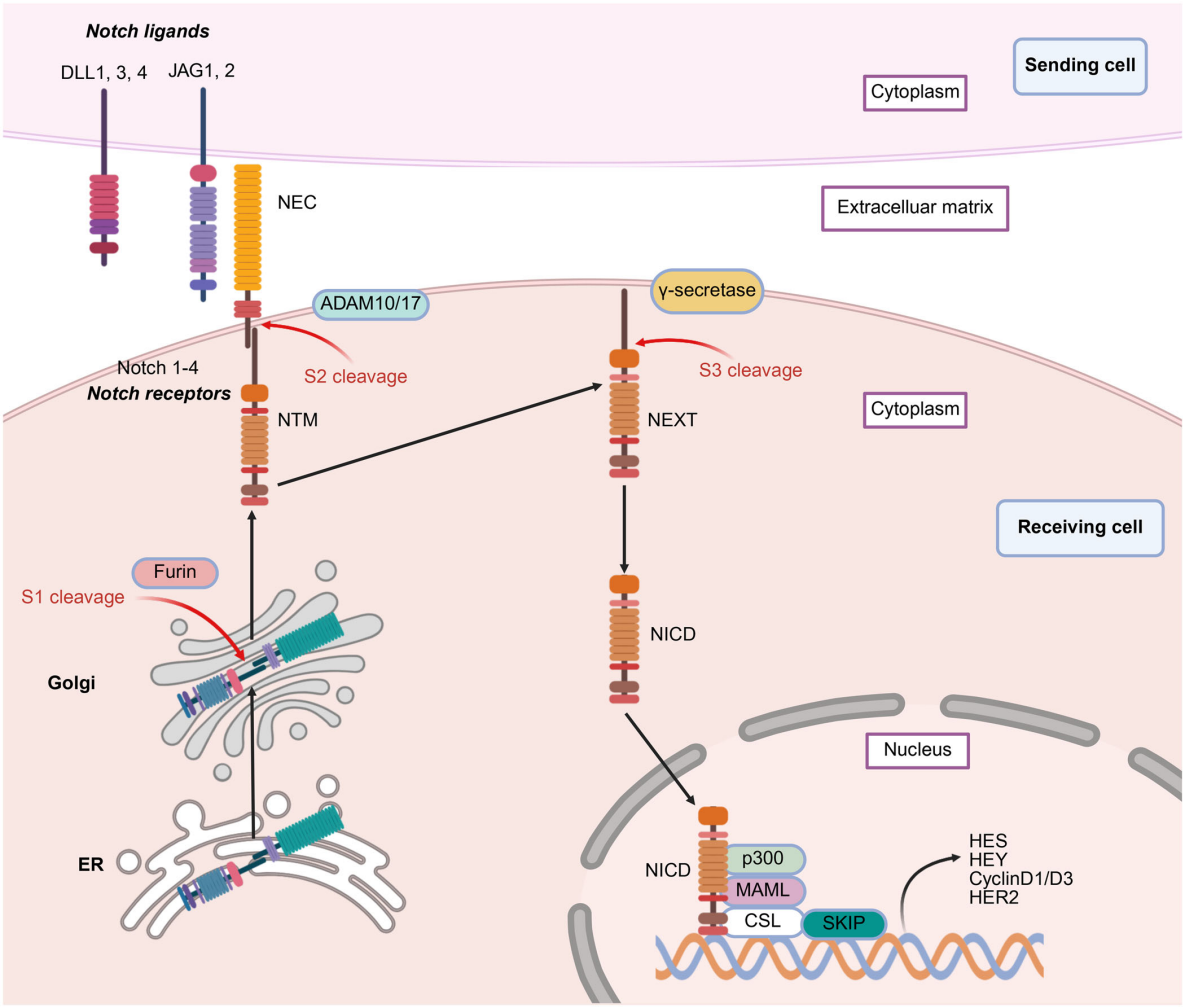
The canonical Notch signaling pathway. The Notch pathway includes four Notch receptors (Notch1, 2, 3, and 4) and five ligands (Jagged1, Jagged2, Dll1, Dll3, and Dll4). Notch receptors are synthesized as inactive precursors and processed by a furin‐like convertase (S1 cleavage) to form a heterodimer. Ligand binding induces ADAM‐mediated S2 cleavage, releasing the extracellular domain, followed by γ‐secretase‐mediated S3 cleavage, which liberates the Notch intracellular domain (NICD). NICD translocates to the nucleus, binds the transcription factor CSL, and recruits co‐activators (MAML, SKIP, and p300) to activate downstream target genes including HES, HEY, cyclin D, and HER2, thereby regulating cell fate, proliferation, differentiation, and survival. Created in https://BioRender.com.

In the field of carcinoma biology, the Notch signaling pathway exhibits significant dual regulatory properties, acting both as a promoter of cancer and, in certain circumstances, as an inhibitor of tumor development. This “double‐edged sword” effect is highly dependent on the specific cellular environment, Notch receptor/ligand subtypes, and tumor type [28]. Research has shown that abnormal activation of the Notch pathway is closely related to the progression of various malignant tumors. The mechanism involves multiple aspects, including the induction of EMT, tumor angiogenesis, maintenance of cancer stem cell characteristics, and remodeling of the immune microenvironment [28, 29].

In ovarian carcinoma, excessive activation of the Notch signaling pathway has been shown to be significantly associated with tumor development, chemotherapy resistance, and poor prognosis [30, 31]. Research has found that the expression patterns of Notch receptors and ligands exhibit high heterogeneity in ovarian carcinoma. Compared with normal ovarian tissue, the expression levels of Notch1, Notch3, and the ligand Jagged1 are significantly upregulated in ovarian carcinoma tissue [32]. It has been confirmed that the Notch signaling pathway is involved in the malignant progression of ovarian carcinoma through multiple mechanisms. For example, Gorji‐Bahri et al. found that the glycoprotein cartilage oligomeric matrix protein (COMP) in the extracellular matrix of ovarian carcinoma cells can activate the Notch3 receptor by enhancing the Notch3–Jagged1 interaction, thereby inducing EMT in ovarian carcinoma cells and promoting tumor invasion and metastasis [33]. Besides, the Notch signaling pathway maintains the stem cell characteristics of ovarian carcinoma cells and enhances their self‐renewal capacity and resistance to treatment by regulating the expression of tumor stem cell markers such as NaNog homeobox (NANOG), octamer‐binding transcription factor 4 (OCT4), cluster of Differentiation 44 (CD44), and cluster of Differentiation 117 (CD117) [34, 35, 36]. What is more, the cross talk between the Notch signaling pathway and other key oncogenic pathways further increases its regulatory complexity in ovarian carcinoma. Studies have shown that the Notch signaling pathway can form positive or negative feedback regulatory loops with multiple signaling pathways, such as PI3K/AKT, NF‐κB, and Wnt/β‐catenin, collectively forming a sophisticated and complex regulatory network [37, 38, 39]. For example, the synergistic activation of Notch signaling and the Wnt/β‐catenin pathway promotes the proliferation and migration of ovarian carcinoma cells [40]. This makes the Notch pathway an extremely attractive target for intervention in ovarian carcinoma treatment.

It can thus be seen that the Notch signaling pathway plays a significant role in the development of ovarian carcinoma. In addition, research has found that this pathway can also regulate the immune microenvironment of ovarian carcinoma, affecting tumor proliferation and metastasis.

#### 2.1.2 The Role of the Notch Signaling Pathway in Regulating the Immune Microenvironment of Ovarian Carcinoma

The TME of ovarian carcinoma has highly immunosuppressive characteristics, which largely mediate tumor immune escape and resistance to immunotherapy [41, 42]. In recent years, an increasing number of studies have revealed the key role of the Notch signaling pathway in shaping the immunosuppressive microenvironment, particularly its mechanism of influencing anti‐tumor immune responses by regulating the functional status of various immune cells.

Tumor‐associated neutrophils (TANs) and regulatory T cells (Tregs) play complex and critical roles in the immune microenvironment of ovarian carcinoma. Studies have shown that TANs have dual regulatory properties. On the one hand, they promote tumor progression through angiogenesis and T cell suppression, on the other hand, they have the plasticity to transform into an anti‐tumor phenotype [43, 44]. Tregs can suppress antitumor immune responses by secreting immunosuppressive factors, thereby promoting tumor development [45]. In addition, Tregs play a central role in immune escape in ovarian carcinoma [46]. A recent study found that JAG2 + TAN cells interact with the Notch1 receptor on the surface of T cells via the JAG2 ligand, activating downstream RBPJ‐dependent transcriptional programs that drive the production of immunosuppressive eTreg cells [47]. This discovery is the first to link three key elements, namely, neutrophils, Notch signaling, and Tregs, and establishes the concept of the “JAG2 + TAN‐Notch1‐eTreg” immune suppression axis.

The Notch signaling pathway also participates in the regulation of other immune cells through multiple mechanisms. Tumor‐associated macrophages (TAMs) are a type of cell that plays an important role in the TME. TAMs include pro‐tumor M2‐type and anti‐tumor M1‐type cells [48]. M2‐type cells promote tumor progression through processes such as mediating angiogenesis and immune suppression [49]. Research indicates that Notch activation promotes its polarization toward the M2 type, enhancing the secretion of immunosuppressive factors [50]. In myeloid‐derived suppressor cells (MDSCs), Notch signaling enhances the inhibitory function of T cell by upregulating reactive oxygen species (ROS) and arginase 1 (ARG1) expression, whereas in dendritic cells (DCs), Notch regulation affects their maturation status and antigen presentation capacity [51, 52].

Hence, the Notch signaling pathway promotes the formation of an immunosuppressive microenvironment in ovarian carcinoma by regulating various types of immune cells, ultimately facilitating the proliferation and metastasis of ovarian carcinoma cells.

#### 2.1.3 Clinical Targeting of the Notch Signaling Pathway

As our understanding of the role of the Notch signaling pathway in ovarian carcinoma deepens, various targeted intervention strategies are being translated from the laboratory to the clinical setting, offering new possibilities for improving the efficacy of ovarian carcinoma treatment. These strategies include small molecule inhibitors, antibody drugs, combination therapy regimens, and biomarker‐based precision therapy, targeting different stages of the Notch signaling pathway to intervene in its abnormal activation and downstream effects.

##### 2.1.3.1 Small Molecule Inhibitors

In the development of small molecule inhibitors, compounds targeting different nodes of the Notch signaling pathway each have their own characteristics.

Gamma‐secretase inhibitors (GSIs) are the first broad‐spectrum Notch pathway‐targeting inhibitors to be developed. GSIs inhibit Notch receptor activation by blocking the final cleavage step of gamma‐secretase, thereby completely blocking all Notch signaling. As a result, GSIs inhibit the proliferation of ovarian carcinoma cells and enhance their sensitivity to platinum‐based drugs. Representative drugs such as LY3039478 have demonstrated efficacy in inhibiting tumor growth and enhancing chemotherapy sensitivity in preclinical ovarian carcinoma models [47]. In addition, Groeneweg et al. pointed out that GSI combined with paclitaxel treatment can inhibit the proliferation and progression of platinum‐resistant ovarian carcinoma [53]. Therefore, therapies targeting the Notch pathway have important clinical value in the initial treatment and recurrence stages of ovarian carcinoma [54]. However, the widespread inhibition of the Notch pathway by GSI often leads to dose‐limiting side effects such as gastrointestinal toxicity, which to some extent limits its clinical application [24, 55]. To overcome this problem, intermittent dosing regimens or local delivery systems need to be developed to maintain antitumor activity while reducing toxicity.

The development of selective Notch inhibitors is another important direction. Studies have shown that targeting Notch3 can increase tumor sensitivity to paclitaxel (PTX) and platinum‐based drugs [56]. Xu et al. found that the multi‐target tyrosine kinase inhibitor (TKI) anlotinib can significantly enhance the sensitivity of ovarian carcinoma cells to cisplatin by inhibiting Notch2 expression and ovarian carcinoma cell stemness characteristics [57]. In this study, anlotinib reduced the expression of Notch2 and stem cell markers OCT4 and NANOG in ovarian carcinoma cells and inhibited tumor spheroid formation. Additionally, anlotinib showed synergistic antitumor effects when combined with cisplatin. These findings provide insights into the repurposing of old drugs and suggest that different Notch subtypes may have unique functions in ovarian carcinoma. Further research is needed to explore this topic in greater depth.

In addition to the aforementioned inhibitors, interventions targeting downstream effectors of the Notch signaling pathway may represent a relatively novel strategy. For example, compounds that target components of the Notch transcription complex or regulate Notch receptor glycosylation or membrane localization may offer more precise control of Notch signaling. These approaches may overcome the limitations of existing Notch‐targeting drugs, but their feasibility requires extensive basic and clinical research to confirm.

##### 2.1.3.2 Specific Antibodies Targeting Notch Ligands or Receptors

Aside from small‐molecule inhibitors, antibody drugs targeting specific components of the Notch pathway have also demonstrated promising therapeutic potential. Since different Notch receptors and ligands play non‐redundant roles in ovarian carcinoma, specifically targeting pathogenic subtypes, such as JAG2 or Notch3, can improve treatment accuracy and reduce side effects. In preclinical studies, anti‐JAG2 antibodies effectively blocked JAG2 + TAN‐mediated eTreg differentiation, restored CD8^+^ T cell function, and did not induce the gastrointestinal toxicity commonly associated with GSI [47, 58].

Intervention targeting the Notch3 receptor is another promising direction. As previously mentioned, COMP can activate the Notch3 signaling pathway by enhancing the Notch3–Jagged1 interaction, driving EMT and metastasis in ovarian carcinoma. Therefore, anti‐Notch3 antibodies may effectively block this pathway, inhibiting tumor invasion and spread [59]. Currently, several anti‐Notch3 antibody drugs have been developed, such as tarextumab (OMP‐59R5). Ovarian carcinoma, particularly subtypes with high COMP expression, may become one of the key indications [60, 61].

Exactly, the combined use of antibody drugs is also worth exploring. Targeted bispecific antibodies may synergistically inhibit the proliferation and metastasis of ovarian carcinoma, but this hypothesis requires further research to confirm.

##### 2.1.3.3 Combined Use of Notch Inhibitors and Immunotherapy

Based on an in‐depth understanding of the mechanism by which the Notch signaling pathway regulates the immune microenvironment, the combination of Notch inhibitors and immune checkpoint inhibitors has emerged as a promising strategy for overcoming immune therapy resistance in ovarian carcinoma.

Research has confirmed that in high‐grade serous ovarian cancer (HGSOC), JAG2 + TANs induce immune‐suppressive eTreg differentiation through Notch signaling, which is one of the key mechanisms underlying the limited efficacy of PD‐1/PD‐L1 inhibitors as monotherapy [47]. In this study, the combination of the Notch inhibitor LY3039478 or anti‐JAG2 monoclonal antibody with a PD‐1 blocker demonstrated significant synergistic effects. The results indicated that the tumor‐infiltrating eTreg proportion was significantly reduced in the combination therapy group, while CD8^+^ T cell infiltration and activity were enhanced. Importantly, the tumor growth inhibition rate was significantly higher than that in the monotherapy group. This combination therapy strategy, by simultaneously alleviating Notch‐mediated innate immune suppression and PD‐1‐mediated adaptive immune suppression, holds promise for breaking through the dual barriers of the ovarian carcinoma immune microenvironment.

Therefore, the combined use of Notch inhibitors and immunotherapy is a promising approach to improve the efficacy of ovarian carcinoma treatment, but further research is still needed to confirm its effectiveness.

As can be seen from the above, targeting the Notch signaling pathway is of great significance in the treatment of ovarian carcinoma, as it plays a key role in tumor development, chemotherapy resistance, and metastasis. Some preclinical animal experiments and clinical trials have confirmed that Notch pathway inhibitors, either alone or in combination with immunotherapy, can inhibit tumor cell growth, enhance chemotherapy sensitivity, and reverse the immunosuppressive microenvironment (**Table** **1**). Despite challenges such as dose‐related toxicity, targeting the Notch pathway offers a new direction for improving the efficacy of treatment for refractory ovarian carcinoma.

**TABLE 1:** Selected clinical and preclinical trials of Notch pathway inhibitors, used alone or in combination with immunotherapy for ovarian carcinoma, spanning the 15‐year period from 2010 to 2025.

Category	Study type	Model/design	Intervention	Result
Preclinical [47]	In vitro + In vivo	Xenograft mice and PDTO models	Anti‐PD‐1 antibody + LY3039478	The efficacy of anti‐PD‐1 monoclonal antibodies is enhanced by the use of LY3039478 or JAG2 neutralizing antibodies targeting Notch signaling.
Preclinical [53]	In vivo	Xenograft mice	GSI, paclitaxel	The combination of GSI and paclitaxel is more effective than either drug used alone in platinum‐resistant ovarian tumors.
Preclinical [57]	In vitro + In vivo	Human ovarian‐cancer cell lines (SKOV3, OVCAR8) + SKOV3 xenograft mice	Anlotinib	Anlotinib inhibits cancer stem cell phenotypes by downregulating NOTCH2 expression.
Clinical [61]	Phase I trial (NCT01277146)	Patient cohorts (*n* = 42)	Tarextumab (OMP‐59R5)	Disease stabilization was seen in patients.
Clinical [62]	Phase II trial (NCT05348356)	Patient cohorts (*n* = 53)	Nirogacestat	Pending

### 2.2 HER2 Signaling Pathway

In recent years, the role of the HER2 signaling pathway in the development, metastasis, and treatment resistance of ovarian carcinoma has attracted increasing attention. As an important member of the epidermal growth factor receptor (EGFR) family, abnormal activation of HER2 can promote tumor cell proliferation, survival, invasion, and metastasis through multiple downstream signaling pathways.

#### 2.2.1 The Biological Characteristics of HER2 and its Expression in Ovarian Carcinoma

HER2, also known as ERBB2, is a 185 kD transmembrane receptor tyrosine kinase composed of an extracellular domain (ECD), a lipophilic transmembrane region, and an intracellular domain (ICD) with tyrosine kinase activity [63, 64]. It is an important member of the EGFR family [65]. The EGFR family includes four closely related receptors, namely, EGFR (HER1/ERBB1), HER2 (ERBB2), HER3 (ERBB3), and HER4 (ERBB4) [66, 67]. Unlike other family members, HER2 has no known direct ligand, and its activation primarily occurs through two mechanisms [68]. The first is the formation of heterodimers with other family members, and the second is through proteolytic cleavage of the ECD protein [69, 70]. HER2 activation initiates multiple downstream signaling pathways, including the Ras/Raf/MAPK signaling pathway, PI3K/Akt/mTOR signaling pathway, and JAK/STAT signaling pathway, thereby regulating key biological processes such as cell proliferation, differentiation, migration, survival, and angiogenesis [71, 72].

In ovarian carcinoma, HER2 expression exhibits significant heterogeneity. Notably, HER2 expression may also vary significantly among different histological types of ovarian carcinoma, with HER2 overexpression being relatively uncommon in clear cell ovarian carcinoma compared to other types [73]. Research indicates that HER2 overexpression is closely associated with the malignant progression of ovarian carcinoma. Patients with HER2 overexpression may have a higher clinical stage and more frequent lymph node metastasis [74, 75]. In addition, HER2 expression status also has important prognostic value. Patients with HER2 overexpression typically exhibit shorter PFS and overall survival, making it a potential marker for predicting poor prognosis in ovarian carcinoma patients [76, 77].

The evolution of HER2 testing methods also reflects a deepening understanding of the disease. Several studies have reported significant differences in the rate of HER2 overexpression among ovarian carcinoma patients across different studies, with some of these differences stemming from inconsistencies in testing methods and evaluation criteria [78, 79]. With the advancement of technology, HER2 protein expression levels are currently mainly detected through immunohistochemistry (IHC), while fluorescence in situ hybridization (FISH) is used to detect HER2 gene amplification status [80, 81, 82, 83]. However, the detection of HER2 in ovarian carcinoma presents unique challenges. A study by Carvajal‐Hausdorf et al. suggested that approximately 42% of patients with serous ovarian carcinoma exhibit high HER2 ICD levels and low ECD levels [84]. This unique expression pattern may cause traditional detection methods targeting the ECD to underestimate the actual activation status of HER2 and partially explains why monoclonal antibodies targeting the HER2 ECD, such as trastuzumab, have limited efficacy in some ovarian carcinoma patients. This finding suggests that targeted therapies against HER2 ICD, such as small molecule TKIs, or in combination with ECD‐targeted drugs, may provide more comprehensive signal blockade for these patients.

#### 2.2.2 Downstream Effects Produced by Activation of the HER2 Signaling Pathway in Ovarian Carcinoma

The HER2 signaling pathway plays a complex and critical role in the development and treatment resistance of ovarian carcinoma. When HER2 is activated, it exerts its oncogenic effects primarily through core downstream pathways such as the Ras/Raf/MAPK pathway, PI3K/Akt/mTOR pathway, STAT signaling pathway, and p38MAPK‐MMP‐2 pathway [85, 86, 87] (Figure 2).

**FIGURE 2:**
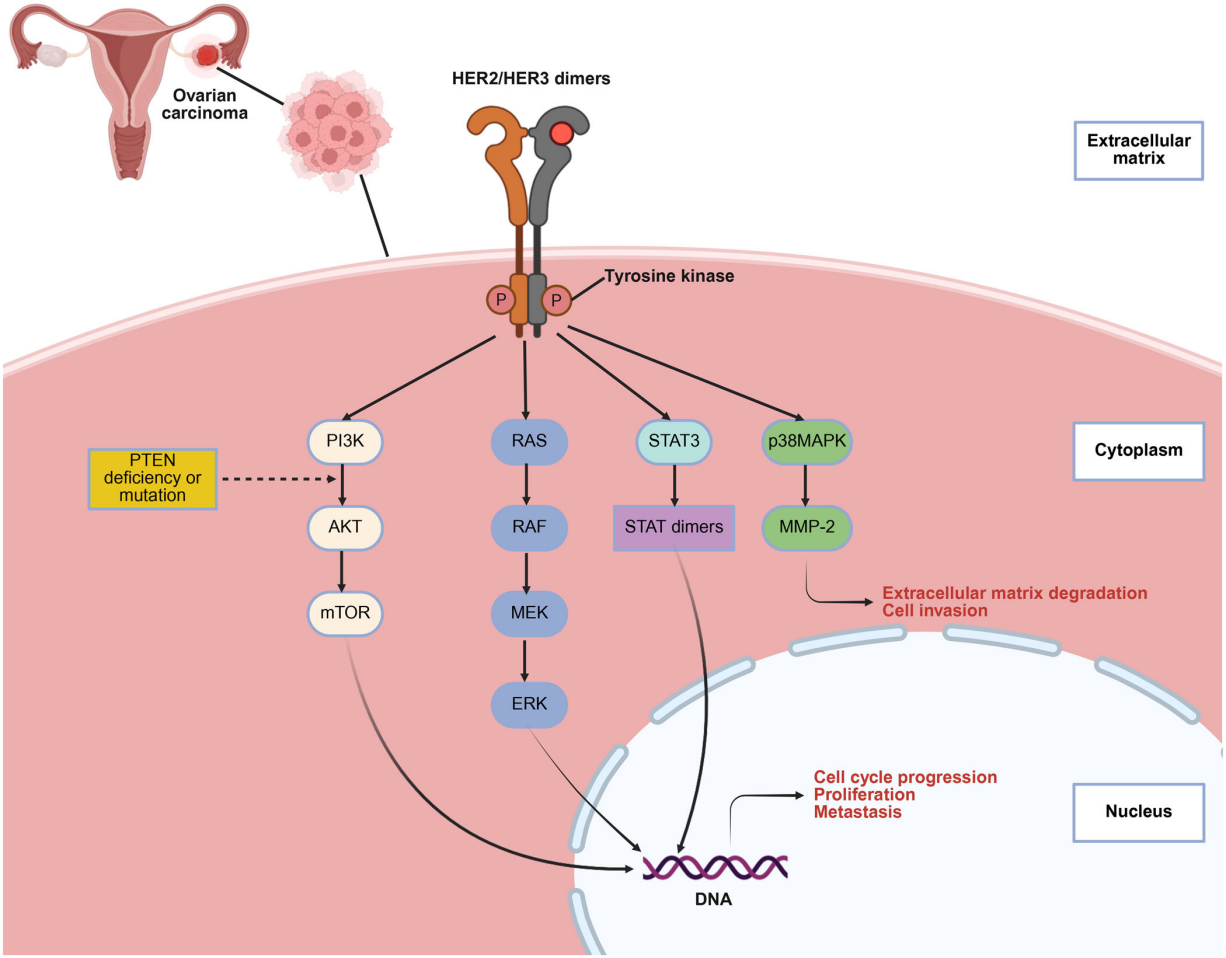
HER2 signaling pathway and downstream oncogenic effects. Upon activation through heterodimerization or ligand‐independent mechanisms, HER2 triggers multiple downstream cascades including the Ras/Raf/MAPK pathway (promoting cell cycle progression and proliferation), the PI3K/AKT/mTOR pathway (enhancing cell survival and inhibiting apoptosis), the STAT signaling pathway (regulating gene transcription), and the p38MAPK‐MMP‐2 pathway (facilitating invasion and metastasis). These pathways collectively drive tumor cell proliferation, survival, and metastatic spread. Created in https://BioRender.com.

The Ras/Raf/MAPK pathway is one of the main pathways for HER2 signal transduction. After HER2 is activated, it promotes the activation of Ras GTPase through the adaptor proteins GRB2 and SOS, which in turn sequentially activate kinases such as Raf, MEK, and ERK [88, 89]. Activated ERK translocates into the cell nucleus, where it regulates the activity of various transcription factors, ultimately promoting cell cycle progression and proliferation [90, 91]. In ovarian carcinoma, HER2 overexpression significantly enhances the activity of the MAPK signaling pathway, which not only accelerates tumor cell proliferation but is also closely related to chemotherapy resistance [92, 93]. Research has shown that sustained activation of the HER2‐mediated MAPK pathway can lead to ovarian carcinoma cells developing resistance to commonly used chemotherapy drugs such as paclitaxel [94].

The PI3K/Akt/mTOR pathway is another key signaling pathway regulated by HER2. After activation, HER2 directly binds to or recruits PI3K, catalyzing the conversion of PIP2 to PIP3, thereby activating PDK1 and Akt [95, 96]. Activated Akt inhibits apoptosis and promotes cell survival by phosphorylating multiple downstream target proteins, such as mTOR [97]. In ovarian carcinoma, HER2 overexpression is often accompanied by the loss or mutation of PTEN, which is an important tumor suppressor gene that negatively regulates the PI3K/Akt pathway, further amplifying Akt signaling and thereby promoting tumor cell survival and drug resistance [98, 99, 100].

The STAT signaling pathway is also indispensable in HER2‐driven ovarian carcinoma progression. Activation of HER2 can phosphorylate STAT family proteins, especially STAT3, promoting the formation of STAT dimers and their translocation to the cell nucleus, where they regulate the expression of specific target genes, ultimately promoting the proliferation and metastasis of ovarian carcinoma cells [101, 102].

The p38MAPK‐MMP‐2 pathway mediates the process by which HER2 promotes tumor invasion and metastasis. Studies have shown that HER2 activation can upregulate p38MAPK activity, thereby increasing the expression of matrix metalloproteinase MMP‐2, which ultimately leads to extracellular matrix degradation and enhanced invasive capacity of ovarian carcinoma cells [103, 104, 105]. Experiments have confirmed that inhibiting HER2 can significantly reduce the MMP‐2/TIMP‐2 ratio and decrease the migration and invasive potential of ovarian carcinoma cells [105].

As can be seen from the above, the HER2 signaling pathway can promote the growth and invasion of ovarian carcinoma by regulating multiple downstream signaling pathways. HER2 overexpression may be closely associated with an unfavorable prognosis in ovarian carcinoma patients.

#### 2.2.3 Clinical Therapeutics Targeting HER2: Monoclonal Antibodies, ADCs, and TKIs

##### 2.2.3.1 Monoclonal Antibody Therapy

Trastuzumab is the first targeted drug approved for the treatment of HER2‐positive breast carcinoma. Trastuzumab works by binding to the ECD IV of HER2, inhibiting downstream signal activation, and inducing antibody‐dependent cell‐mediated cytotoxicity (ADCC) [106, 107, 108, 109, 110]. To date, numerous studies have confirmed the efficacy of trastuzumab in the treatment of ovarian carcinoma. For example, Cong et al. pointed out that trastuzumab can inhibit tumor growth by blocking cell proliferation and promoting apoptosis, providing a potential new strategy for ovarian carcinoma treatment [111]. In addition, a Phase I trial found that a patient with ovarian cancer experienced complete resolution of ascites and pleural effusion after receiving trastuzumab in combination with alvespimycin hydrochloride, which persisted for 24.8 months [112]. However, compared with breast carcinoma, trastuzumab has relatively limited efficacy in ovarian carcinoma, with a lower overall response rate (ORR) when used as monotherapy. This may be related to the fact that HER2 expression levels in ovarian carcinoma are generally lower than those in breast carcinoma [113]. In a phase II trial, Bookman et al. demonstrated that trastuzumab monotherapy has limited efficacy in recurrent ovarian cancer due to the rarity of HER2 overexpression and low rate of objective response among patients with HER2 overexpression [114].

Pertuzumab is another HER2‐targeted monoclonal antibody that binds to the ECD II of HER2 and primarily inhibits the dimerization of HER2 with other members of the ERBB family, particularly HER3 [115, 116, 117]. Previous studies have indicated that pertuzumab is a viable treatment option for ovarian carcinoma [118]. For example, Agus et al. confirmed in a Phase I clinical study that pertuzumab may be a viable anti‐cancer strategy by inhibiting dimerization [119]. Additionally, Gordon et al. noted that pertuzumab was well‐tolerated in patients with advanced refractory ovarian carcinoma [120]. Results from another study suggest that the objective response rate in the gemcitabine plus pertuzumab group was 13.8%, significantly higher than the 4.6% in the placebo control group. Therefore, pertuzumab may enhance the efficacy of gemcitabine in platinum‐resistant ovarian carcinoma [121]. However, a phase II trial indicated that adding pertuzumab to carboplatin‐based chemotherapy failed to significantly improve PFS in platinum‐sensitive ovarian cancer patients overall [122]. Compared with trastuzumab, pertuzumab may be more advantageous in HER2‐low tumors, as its inhibition of HER2 dimerization is not entirely dependent on HER2 expression levels [123].

The strategy of combining targeted drugs provides direction for improving the efficacy of monoclonal antibodies. Preclinical studies have shown that the combination of trastuzumab and pertuzumab produces a synergistic effect, exhibiting stronger growth inhibition than either drug alone in both HER2‐high and HER2‐low ovarian cancer cells [124]. This is because the two antibodies target different epitopes of HER2, achieving more comprehensive signal blockade [123]. In a case report, a patient with stage IV HGSOC with ErbB2 amplification received trastuzumab–pertuzumab combination therapy after chemotherapy resistance, but without concomitant chemotherapy. The results showed that the metastatic lesions had shrunk after 36 months, demonstrating the long‐term benefit potential of dual‐targeted therapy [125].

In summary, monoclonal antibodies, either alone or in combination with other therapies, can exert their therapeutic effects by targeting HER2 on the surface of ovarian cancer cells, and this has been confirmed in some trials (Table 2). Monoclonal antibodies may improve the precision treatment landscape for ovarian carcinoma. With advances in technology, ADCs are also showing promise in the treatment of ovarian carcinoma.

**TABLE 2:** Selected clinical trials of anti‐HER2 agents (trastuzumab and pertuzumab) in ovarian carcinoma, published from 2000 to 2025.

Drug	Disease	Patient	Result	Status/phase
Trastuzumab [111]	Epithelial ovarian cancer	60	The relative expression level of HER2 mRNA in epithelial ovarian tumor tissue is 55 times higher than that in normal ovarian tissue. Trastuzumab significantly inhibits the proliferation of SKOV3 cells.	Completed
Trastuzumab [112]	Persistent or recurrent epithelial ovarian or primary peritoneal carcinoma	45	The ORR was 7.3%, with a median treatment duration of 8 weeks and a median progression‐free interval of 2 months.	Phase II
Trastuzumab and alvespimycin hydrochloride [114]	Advanced solid tumors	28 (25, breast; 3, ovarian)	An ovarian cancer patient had complete resolution of ascites and pleural effusion that lasted 24.8 months.	Phase I
Pertuzumab [119]	Advanced, recurrent or metastatic solid tumors	21	A patient with ovarian cancer (5.0 mg/kg) achieved PR.	Phase I
Pertuzumab [120]	Advanced, refractory OC	61 (cohort 1), 62 (cohort 2)	Pertuzumab is well tolerated with a RR of 4.3% in heavily‐pretreated OC patients.	Phase II
Pertuzumab + gemcitabine versus placebo + gemcitabine [121]	Advanced, platinum‐resistant epithelial ovarian, fallopian tube, or primary peritoneal cancer	130 (65 per arm)	ORR 13.8% in pertuzumab + gemcitabine vs. 4.6% in placebo + gemcitabine.	Completed
Pertuzumab + Trastuzumab [122]	FIGO stage IV high‐grade serous ovarian cancer with an amplification of ERBB2	1	Present a long‐lasting PR after 36 months.	Completed
Carboplatin‐based chemotherapy with or without pertuzumab [125]	Relapsed, platinum‐sensitive ovarian cancer	149	PFS 34.1 months in chemotherapy plus pertuzumab arm vs. 37.3 months in chemotherapy‐only arm.	Phase II

##### 2.2.3.2 ADCs

ADCs consist of three key components, namely, a monoclonal antibody that targets a specific antigen, a cytotoxic drug that kills cells, and a chemical linker that covalently binds the two together [126]. ADCs combine targeting with cytotoxicity by using antibodies to specifically deliver potent cytotoxic drugs to HER2‐positive tumor cells, significantly improving the therapeutic index [127]. In recent years, various anti‐HER2 ADCs have demonstrated breakthrough efficacy in the treatment of ovarian carcinoma.

Trastuzumab emtansine (T‐DM1) is an ADC that conjugates trastuzumab with the microtubule inhibitor DM1, which is a mitoxantrone derivative [128]. Its mechanism of action begins with binding to the HER2 receptor, forming a complex that is internalized through receptor‐mediated endocytosis [129, 130]. The drug's unique irreversible linker design keeps it stable in the bloodstream and TME, releasing active DM1 only after partial degradation by antibodies in lysosomes [131]. Free DM1 metabolites inhibit microtubule polymerization, ultimately inducing apoptosis in target cells [132]. Currently, T‐DM1 has been approved for use in HER2‐positive breast carcinoma [133, 134]. In addition, T‐DM1 is gradually being used in ovarian carcinoma. Studies have shown that T‐DM1 exhibits significant tumor‐suppressing effects in HER2‐overexpressing ovarian carcinoma [135, 136]. However, T‐DM1 treatment of tumors may lead to drug resistance and has certain toxicity issues, such as thrombocytopenia and hepatotoxicity [137, 138, 139].

Trastuzumab deruxtecan (T‐DXd, DS‐8201a) is an ADC targeting HER2, consisting of trastuzumab, a cleavable GGFG peptide linker, and a topoisomerase I inhibitor (DXd) [140, 141]. After entering tumor cells, T‐DXd releases DXd, a small molecule that can penetrate neighboring cell membranes and exert an antitumor effect by inducing DNA damage [142]. T‐DXd has been approved for the treatment of adult patients with unresectable or metastatic HER2‐positive solid tumors who have received systemic therapy but lack effective alternative treatment options [143]. Surprisingly, T‐DXd is also effective against tumors with low HER2 expression [144]. The application of T‐DXd in ovarian carcinoma is currently being explored. In a case study by Kong et al., a patient with HER2‐positive ovarian clear cell carcinoma who received T‐DXd treatment exhibited a unique clinical response. Therefore, further experimental studies are needed to explore the potential therapeutic value of this drug in ovarian clear cell carcinoma [145]. Another study indicated that T‐DXd has the potential to become an effective ADC for the treatment of primary and metastatic HER2‐overexpressing HGSOC and OCCC [146].

Trastuzumab duocarmazine (SYD985) is a novel HER2‐targeted ADC composed of the anti‐HER2 monoclonal antibody trastuzumab conjugated with the cytotoxic drug duocarmazine, capable of specifically targeting HER2‐positive tumor cells [147]. This drug combines trastuzumab with docetaxel to specifically recognize and bind to DNA grooves, thereby producing a unique cytotoxic effect and inducing tumor cell death [148]. Nadal‐Serrano et al. pointed out that SYD985 could be tried for the treatment of tumors resistant to T‐DM1 [149]. Additionally, SYD985 may also exhibit antitumor activity in tumors with low HER2 expression. But further research is required to confirm this. Currently, some studies are focusing on the use of SYD985 for the treatment of ovarian carcinoma with HER2 expression. Preclinical studies have demonstrated that SYD985 exhibits significant antitumor activity in ovarian carcinoma sarcomas with HER2/Neu expression [150]. Surprisingly, SYD985 can also inhibit the progression of epithelial ovarian carcinoma with HER2/Neu expression [151]. Currently, clinical data on SYD985 in ovarian carcinoma are still in the early stages, but preliminary results show promising prospects. It is speculated that SYD985 may offer a new treatment option for patients with HER2‐positive ovarian carcinoma.

Additionally, there are some newly developed ADCs, such as RC48 and JSKN003, which may hold promise for the treatment of HER2‐positive ovarian carcinoma. RC48 is an ADC whose structure includes a humanized anti‐HER2 monoclonal antibody, hertuzumab, covalently linked via a cleavable linker to the cytotoxic drug monomethyl auristatin E (MMAE) [152]. Some scholars have suggested that RC48 can inhibit the development of various types of malignant tumors, such as ovarian carcinoma and breast carcinoma [153]. In addition, JSKN003, as a HER2 bispecific ADC, may offer a new treatment option for platinum‐resistant ovarian carcinoma [154, 155, 160].

ADC shows great promise in the treatment of ovarian carcinoma by targeting cytotoxic drugs to tumor cells, thereby improving efficacy and reducing systemic toxicity. Currently, some ADCs have demonstrated antitumor activity in clinical trials, particularly showing potential value for patients with platinum resistance. In the future, with optimized targets, improved linker technologies, and the exploration of combination strategies (such as co‐administration with PARP inhibitors or immunotherapy), ADCs are expected to become an important option for precision treatment of ovarian carcinoma.

##### 2.2.3.3 Small Molecule TKIs

TKIs are a class of anticarcinoma drugs that target and inhibit tyrosine kinase activity [156]. It has been proven that TKIs inhibit tumor cell proliferation, invasion, and angiogenesis by blocking abnormally activated kinase signaling pathways, such as EGFR and vascular endothelial growth factor receptor (VEGFR) [157]. In HER2‐positive ovarian carcinoma, small molecule TKIs block downstream signal transduction by inhibiting HER2 tyrosine kinase activity [158, 159].

Lapatinib is a reversible dual EGFR/HER2 TKI and a promising drug for the treatment of breast carcinoma [160, 161]. In ovarian carcinoma, lapatinib can enhance the sensitivity of ovarian carcinoma cells to carboplatin by inhibiting HECTD3 expression [162]. Therefore, the combination of lapatinib and carboplatin can significantly improve antitumor efficacy, providing a new treatment option for serous ovarian carcinoma. Besides, lapatinib can also enhance the chemotherapy sensitivity of paclitaxel‐resistant cells [163, 164].

Afatinib and neratinib are irreversible pan‐HER family inhibitors that simultaneously target EGFR, HER2, and HER4 [165, 166]. Afatinib is used to treat ovarian carcinoma because it effectively inhibits the proliferation of ovarian carcinoma cells [167]. Neratinib effectively inhibits tumor growth by suppressing the proliferation, signal transduction, and cell cycle progression of epithelial ovarian carcinoma cells with HER2 amplification **[** 168]. Additionally, studies have found that olaparib, a PARP inhibitor, combined with neratinib exhibits synergistic antitumor effects in HER2‐positive ovarian carcinoma [169]. Therefore, the combined use of olaparib and neratinib potentially provides a new treatment strategy for this patient population.

Therefore, small‐molecule TKIs demonstrate significant potential in ovarian carcinoma treatment by targeting and regulating key signaling pathways involved in tumor growth, metastasis, and drug resistance. They can be used as monotherapy or in combination with chemotherapy, PARP inhibitors, and other agents to enhance anti‐tumor activity, particularly offering clinical value for patients with recurrent or drug‐resistant disease. Although some TKIs are still in the research phase, with the advancement of precision medicine, TKIs targeting specific molecular subtypes hold promise for providing ovarian carcinoma patients with more efficient and less toxic treatment options, thereby improving outcomes.

As mentioned above, targeting the HER2 pathway provides an important therapeutic strategy for ovarian carcinoma treatment, particularly for patients with HER2 overexpression or amplification subtypes. As a member of the EGFR family, abnormal activation of HER2 promotes tumor proliferation, metastasis, and chemotherapy resistance. Monoclonal antibody drugs, such as trastuzumab and pertuzumab, significantly inhibit tumor growth by blocking signal transduction or delivering cytotoxic drugs. In addition, several preclinical trials and clinical trials have proposed the possibility of ADC and TKI treatment for ovarian carcinoma (Table 3). HER2‐targeted therapy is expected to become an important option for individualized treatment of ovarian carcinoma.

**TABLE 3:** Selected clinical trials of ADCs and TKIs in ovarian carcinoma, published from 2010 to 2025.

Category	Study type	Model/design	Intervention	Result
Preclinical [135]	In vitro + In vivo	Primary EOC cell lines, xenograft models with 3+ HER2/neu expression	Trastuzumab (T), pertuzumab (P), T+P, trastuzumab‐emtansine (T‐DM1)	Compared to T alone, P alone and T+P, T‐DM1 induces significantly more apoptosis and more effectively inhibits tumor growth in EOC xenograft tumors.
Preclinical [136]	In vivo	Mice bearing intraperitoneal and subcutaneous SKOV3 xenografts	Trastuzumab‐DM1	Subcutaneous xenograft model: T‐DM1 (30 mg/kg) induced long‐term tumor regression without regrowth after treatment. Peritoneal xenograft model: Intraperitoneal T‐DM1 (30 mg/kg) nearly eliminated tumors in 3 of 7 mice.
Clinical [145]	Case report	Recurrent Ovarian Clear Cell Carcinoma	Trastuzumab deruxtecan (T‐DXd)	After three cycles of T‐DXd treatment, a significant reduction in the retroperitoneal and pelvic nodes, splenic lesions, ascites, and pleural effusion.
Preclinical [146]	In vitro + In vivo	Primary ovarian cancer cell lines (7 HGSOC and 4 CC), Mouse models harboring HGSOC and CC xenografts with HER2 3+ expression	T‐DXd, CTL ADC	Compared to CTL ADC, T‐DXd exhibits significantly higher efficacy against HER2 3+ HGSOC and CC cell lines and mouse xenograft models.
Preclinical [150]	In vitro + In vivo	Primary carcinosarcomas cell lines, mouse xenograft and patient‐derived xenograft	SYD985, T‐DM1	Compared to T‐DM1, SYD985 is more effective against cell lines and xenografts expressing HER2/neu.
Preclinical [151]	In vitro + In vivo	Primary EOC cell lines, xenografts	SYD985, T‐DM1	SYD985 demonstrates superior efficacy over T‐DM1 in targeting HER2/neu 3+ cell lines and xenografts.
Clinical [167]	Case report	High grade serous carcinoma	Afatinib	The progression‐free interval is 10 months.
Preclinical [168]	In vitro + In vivo	Primary tumor cell lines and HER2‐amplified epithelial ovarian carcinoma xenografts	Neratinib	Targeting HER2 amplification, neratinib was highly effective in cell lines and consequently prolonged survival in ovarian carcinoma xenografts.
Preclinical [169]	In vitro + In vivo	Ovarian cancer cell lines and xenografts with variable HER2 expression	Neratinib, olaparib	HER2+ OC cell lines demonstrated higher sensitivity to olaparib and neratinib when compared to HER2 negative tumors.
Preclinical [162]	In vitro + In vivo	Serous ovarian cancer cell lines, xenografts	Lapatinib, carboplatin	Lapatinib can enhance the chemosensitivity of ovarian cancer cells to carboplatin by downregulating HECTD3 expression.
Clinical [161]	Phase II trials	Epithelial ovarian cancer with relapse after first line of chemotherapy	Lapatinib + topotecan	ORR, 14%

### 2.3 DNA Damage Repair (DDR) Pathway

The DDR pathway is a key mechanism for maintaining genomic stability. Defects in its function can lead to the accumulation of mutations, chromosomal abnormalities, and malignant cell transformation, thereby promoting tumorigenesis [170, 171]. The DDR pathway mainly includes direct repair (DR), base excision repair (BER), nucleotide excision repair (NER), mismatch repair (MMR), homologous recombination repair (HRR), and so on. Among them, defects in the HRR pathway are closely related to the occurrence of ovarian carcinoma [172, 173, 174, 175, 176].

#### 2.3.1 DDR Mechanics and the Concept of BRCAness

HRR is a highly faithful mechanism for cells to repair double‐strand breaks (DSBs) in DNA [177]. It precisely restores damaged sequences using sister chromatids or homologous chromosomes as templates, thereby maintaining cellular function [178, 179]. HRR relies on core proteins such as RAD51 and BRCA1/2 and is crucial for maintaining genomic stability and anti‐tumor defense [180, 181] (Figure 3). Homologous recombination deficiency (HRD) refers to the loss of HRR function in cells due to gene mutations, such as BRCA1/2 and RAD51 gene mutations, or epigenetic silencing, rendering them unable to effectively repair DSBs in DNA [182, 183]. This concept of “BRCAness” extends the HRD phenotype beyond germline BRCA mutations. HRD leads to genomic instability, accumulation of chromosomal abnormalities, and increased sensitivity to treatments such as PARP inhibitors [184]. These defects are commonly found in malignant tumors such as breast carcinoma, ovarian carcinoma, and prostate cancer and serve as important targets for biomarkers and targeted therapy [185, 186, 187, 188].

**FIGURE 3:**
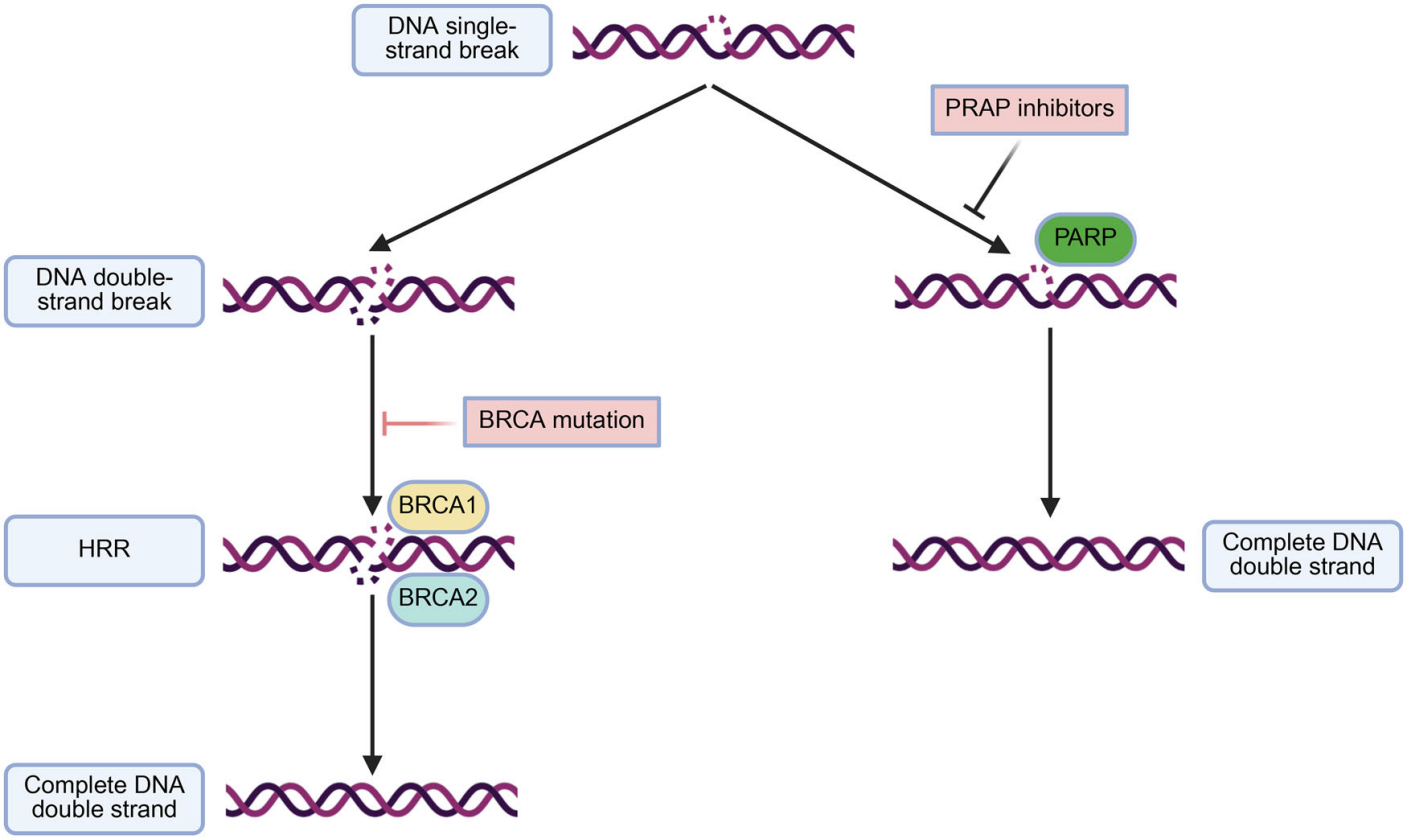
Synthetic lethality of PARP inhibition in HRD tumors. In cells with functional homologous recombination (HR), single‐strand breaks (SSBs) are repaired by PARP, while double‐strand breaks (DSBs) are accurately repaired via the HR pathway involving BRCA1/2. In HR‐deficient (HRD) cells (e.g., due to BRCA mutation), DSBs cannot be faithfully repaired. PARP inhibitors (PARPi) trap PARP on DNA, converting SSBs into DSBs. The combined effect of HRD and PARPi leads to an accumulation of irreparable DSBs and synthetic lethality. Created in https://BioRender.com.

#### 2.3.2 Implications in Ovarian Carcinoma Therapeutics

Numerous studies have explored the relationship between ovarian carcinoma and HRD, revealing that HRR defects are a significant molecular characteristic of ovarian carcinoma, especially HGSC [189, 190]. Data show that HRD can be observed in more than 50% of high‐grade serous ovarian carcinomas, with BRCA1/2 gene mutations being the most characteristic driving factor of HRD [191, 192]. Some studies have pointed out that loss of homologous recombination (HR) function leads to EOC genomic instability and forces tumor cells to rely on PARP‐mediated alternative DNA repair pathways [193] (Figure 3). Therefore, PARP inhibitors can be used to treat ovarian carcinoma with HRR defects. Clinically, HRD testing can not only guide the use of clinical drugs such as PARP inhibitors but also assess patient prognosis.

It can thus be seen that DDR pathways play a key role in ovarian carcinoma, and their dysfunction leads to genomic instability and promotes tumorigenesis. At the same time, DDR defects make ovarian carcinoma sensitive to targeted therapies such as PARP inhibitors, making them an important treatment direction.

#### 2.3.3 Clinical Targeting of DDR: PARP Inhibitors and Combinations

As can be seen from the above, defects in HRR function induce genomic instability in ovarian carcinoma and force cancer cells to rely on PARP‐mediated alternative repair pathways [194]. Therefore, ovarian carcinoma with HRR defects does indeed exhibit sensitivity to PARP inhibitors [195]. The main mechanism is described below. PARP inhibitors block the DNA single‐strand repair pathway in tumor cells by inhibiting PARP activity, particularly BER [196]. When PARP function is inhibited, single‐strand breaks in DNA accumulate and convert into more lethal DSBs [197]. In tumor cells with HRD, genomic instability and cell apoptosis occur due to the inability to repair DSBs through HR. This phenomenon is called “synthetic lethality” [198]. This characteristic makes PARP inhibitors such as olaparib and niraparib important treatment options for HRD‐positive ovarian carcinoma [199].

Currently, an increasing number of studies are focusing on PARP inhibitors, with olaparib and niraparib remaining the most commonly used and effective drugs for ovarian carcinoma. Olaparib, as maintenance therapy, provides significant long‐term survival benefits for patients with BRCA‐mutated advanced ovarian carcinoma. Banerjee et al. reported the follow‐up results of a randomized controlled trial, suggesting that the median PFS (56.0 months) in the olaparib group was significantly longer than that in the placebo group (13.8 months), but olaparib was associated with anemia and neutropenia [200]. DiSilvestro et al. analyzed 7‐year follow‐up data showing that the 7‐year survival rate in the olaparib group was 67.0%, significantly higher than that in the placebo group (46.5%), and olaparib was also found to be safe, with low rates of bone marrow abnormalities and leukemia [201]. These results support the use of olaparib maintenance therapy to prolong remission time and potentially improve the chances of cure. In addition to olaparib, niraparib also plays an important role in patients with advanced ovarian carcinoma. In a randomized, double‐blind phase III trial evaluating the efficacy of niraparib maintenance therapy, results showed that niraparib significantly prolonged PFS in HRD patients (21.9 months) with acceptable safety [202]. In a phase III study, Monk et al. conducted a median follow‐up of 73.9 months and found that maintenance therapy with niraparib did not significantly improve overall survival in patients with advanced ovarian carcinoma but significantly improved long‐term PFS [203]. Surprisingly, the probability of HRD patients remaining progression‐free after 5 years of treatment with niraparib was twice that of the placebo group. Therefore, maintenance treatment with niraparib demonstrated sustained PFS benefits in HRD patients.

However, the emergence of acquired resistance to PARP inhibitors is a major clinical challenge. The mechanisms of resistance include reduced PARP capture, restoration of HR function, and enhanced replication fork protection [204]. Numerous studies have found that BRCA genes can undergo reverse mutations, thereby restoring HRR function [205]. Besides, PARP1‐DOT1L may be one of the causes of acquired resistance to PARP inhibitors. Targeting and inhibiting DOT1L can enhance the therapeutic response of ovarian carcinoma patients to PARP inhibitors, representing a potential effective strategy [206]. In addition, Wu et al. found that there are abnormally activated super‐enhancers (SEs) in ovarian carcinoma, and the SE‐driven transcription factor KLF5 is a key regulator of PARP inhibitor resistance in OC patients [207]. This provides new insights into the treatment of PARP inhibitor‐resistant OC. Although some studies have explored PARP inhibitor resistance, future research should continue to focus on dynamic monitoring of resistance markers and the development of novel combination therapies to expand the patient population benefiting from PARP inhibitors and improve patient outcomes.

In addition to single‐target drugs, ovarian carcinoma treatment is gradually beginning to adopt multi‐pathway combined intervention strategies. For example, anti‐angiogenic drugs, such as bevacizumab, combined with PARP inhibitors can significantly prolong PFS in HRD‐positive patients [208, 209]. Some studies are also investigating the synergistic effects of PD‐L1 inhibitors and PARP inhibitors [210, 211]. In the future, multi‐omics analysis should be used to achieve precise classification. What's more, innovative technologies such as novel biomarkers and dual targets should be used to further optimize individualized treatment.

### 2.4 PI3K/AKT/mTOR Signaling Pathway

#### 2.4.1 Pathway Activation and Genetic Alterations

The PI3K/AKT/mTOR pathway is a central regulator of cell growth, proliferation, and survival. In ovarian carcinoma, this pathway is frequently hyperactivated due to genetic alterations, including PIK3CA activating mutations, PTEN loss or mutation, and AKT amplification [99, 100]. As noted in Section 2.2.2, this pathway is also a key downstream effector of HER2, and their crosstalk is a major driver of tumor progression and drug resistance.

#### 2.4.2 Contribution to Cell Survival and Drug Resistance

Activation of this pathway promotes tumor cell survival by inhibiting apoptosis and is strongly implicated in chemotherapy resistance. It is well‐established that sustained PI3K/AKT/mTOR signaling can lead to ovarian carcinoma cells developing resistance to platinum agents and taxanes. The PI3K/AKT/mTOR pathway also cross talks with other signaling cascades, including the Ras/MAPK pathway and the estrogen receptor pathway, further complicating therapeutic interventions.

#### 2.4.3 Clinical Experience With PI3K/AKT/mTOR Inhibitors

Given its central role, several inhibitors targeting this pathway have entered clinical trials. These include pan‐PI3K inhibitors (e.g., buparlisib), AKT inhibitors (e.g., ipatasertib), and mTOR inhibitors (e.g., everolimus). However, clinical efficacy as monotherapy has been limited, often due to dose‐limiting toxicities and the activation of compensatory feedback loops. For instance, inhibition of mTOR can lead to feedback activation of PI3K/AKT via upstream receptor tyrosine kinases, limiting single‐agent activity. Current research is focused on rational combinations, such as co‐targeting PI3K/mTOR with MEK inhibitors or using them in combination with chemotherapy, to overcome resistance and improve therapeutic index. Biomarker‐driven trials selecting patients with PIK3CA mutations or PTEN loss are also underway to better identify potential responders [212, 213].

### 2.5 Angiogenesis Pathway (vascular endothelial growth factor [VEGF]/VEGFR)

#### 2.5.1 VEGF Signaling in the Ovarian Cancer Microenvironment

Angiogenesis, the formation of new blood vessels, is a hallmark of cancer and is particularly prominent in ovarian carcinoma. The VEGF family and its receptors (VEGFR) are key drivers of this process. In ovarian cancer, VEGF is overexpressed and contributes to ascites formation, tumor growth, and metastatic spread by promoting an aberrant, leaky vasculature [214, 215]. Hypoxia within the TME is a major inducer of VEGF expression, creating a vicious cycle of angiogenesis and tumor progression.

#### 2.5.2 Anti‐angiogenic Therapy: Bevacizumab and Other VEGFR Inhibitors

Bevacizumab, a humanized monoclonal antibody targeting VEGF‐A, was the first anti‐angiogenic therapy approved for ovarian cancer. Clinical trials have demonstrated that adding bevacizumab to carboplatin and paclitaxel, followed by bevacizumab maintenance, significantly improves PFS in patients with newly diagnosed advanced disease. It is also an active agent in the recurrent setting. Other multi‐targeted VEGFR TKIs, such as cediranib and pazopanib, have also shown activity. However, anti‐angiogenic therapy is not without challenges; no validated biomarkers exist to predict response, and resistance inevitably develops, often through upregulation of alternative pro‐angiogenic pathways (e.g., FGF and PDGF). Combination strategies with PARP inhibitors have shown particular promise, as VEGF inhibition can induce hypoxia and impair HRR, potentially sensitizing tumors to PARP inhibition [216, 217].

## 3 Cross talk Between Signaling Pathways and Therapeutic Implications

The signaling pathways described above do not operate in isolation. On the contrary, they form a highly complex and interactive network. For instance, as highlighted, the HER2 pathway directly activates the PI3K/AKT/mTOR and MAPK cascades. Furthermore, Notch signaling can crosstalk with the PI3K/AKT and Wnt/β‐catenin pathways, forming positive or negative feedback loops that collectively regulate tumor cell behavior [37, 38, 39]. Similarly, hypoxia in the TME can simultaneously activate both the VEGF pathway (to drive angiogenesis) and the DDR pathway (by inducing replication stress) [218].

This extensive crosstalk has profound therapeutic implications: inhibiting a single pathway (e.g., HER2 with trastuzumab) may be ineffective if a parallel pathway (e.g., PI3K/AKT/mTOR) is constitutively activated due to a PTEN mutation. This provides a strong mechanistic rationale for combination therapies, such as the co‐administration of PARP inhibitors with anti‐angiogenic agents, where the VEGF inhibitor can create a hypoxic environment that further impairs HR repair, potentially re‐sensitizing tumors to PARP inhibition [219]. Understanding these network interactions is therefore critical for designing effective, multi‐pronged therapeutic strategies [220].

## 4 Challenges and Future Perspectives

### 4.1 Current Challenges in the Field

However, although targeted therapy targeting ovarian carcinoma signaling pathways has made progress, it still has shortcomings, with its core deficiency lying in the intrinsic complexity and adaptive resistance of ovarian carcinoma. First, the high heterogeneity and dynamic evolution of tumors lead to frequent primary and acquired drug resistance, with complex mechanisms involved. For example, in PARP inhibitor therapy, secondary mutations in the BRCA gene may restore the HRR capacity of tumor cells [205]. Second, carcinoma cell signaling networks exhibit extensive cross talk and compensatory feedback loops. When a single pathway is inhibited, it often triggers compensatory activation of alternative pathways, thereby weakening treatment efficacy or even leading to treatment failure. Furthermore, many pathway inhibitors, whose targets also function in normal tissues, are prone to dose‐limiting toxicity, limiting their clinical application. A major challenge remains the lack of reliable predictive biomarkers to stratify patients who will most likely benefit from a given targeted agent. Finally, traditional preclinical models (such as cell lines and patient‐derived xenograft models) cannot fully simulate the complex tumor–immune microenvironment interactions in the human body, significantly hindering the clinical translation efficiency of new drug development.

### 4.2 Novel Therapeutic Directions and the Path Forward

In future research, greater attention should be focused on exploring multidimensional, systematic integrated treatment strategies. The primary direction should be the development of rational combination therapies that simultaneously target multiple key pathway nodes or combine immunotherapy to block compensatory activation, induce synthetic lethality, and reverse immune suppression, thereby achieving synergistic effects. Promising examples include the combination of PARP inhibitors with anti‐angiogenic agents [209], and the pairing of Notch inhibitors with immune checkpoint blockers [47]. Additionally, developing the next generation of highly selective inhibitors to enhance efficacy and reduce toxicity is also crucial. Furthermore, novel strategies such as bispecific antibodies (e.g., targeting both HER2 and other receptors) and novel cellular immunotherapies (e.g., CAR‐T cells targeting MSLN or MUC16) are being actively explored in preclinical and early‐phase clinical studies [221]. Of course, overcoming drug resistance is another area worthy of investigation. By conducting in‐depth research into drug resistance mechanisms to develop reversal strategies, such as compounds targeting BRCA reversal or inhibitors of downstream compensatory pathways (e.g., DOT1L inhibitors to overcome PARPi resistance [206]). Equally important is the development of more advanced preclinical models, such as patient‐derived organoids and humanized mouse models, and the adoption of innovative adaptive clinical trial designs to accelerate the development of new drugs targeting the various molecular subtypes of ovarian carcinoma. The ultimate goal is to translate breakthroughs in basic research into a substantial extension of survival for ovarian carcinoma patients. These development directions collectively form the future research blueprint for targeted therapy in ovarian carcinoma. If successfully implemented, it is expected to significantly improve the clinical prognosis of this challenging carcinoma.

## 5 Conclusion

Ovarian carcinoma remains a lethal malignancy due to its late diagnosis, high heterogeneity, and inevitable drug resistance. This review has detailed the central roles of key signaling pathways—Notch, HER2, DDR, PI3K/AKT/mTOR, and VEGF—in driving these malignant behaviors. The translation of this knowledge into clinical practice, particularly through PARP inhibitors for HRD‐positive tumors and anti‐angiogenic agents, has improved outcomes for a subset of patients. However, the complexity of pathway crosstalk and the rapid onset of acquired resistance demand a shift from single‐agent therapies to rational, biomarker‐guided combination strategies. Future success will depend on a deeper understanding of pathway dynamics, the development of novel agents (e.g., next‐generation ADCs), and the application of sophisticated preclinical models to validate these approaches. Ultimately, a concerted effort to integrate these insights into individualized treatment regimens holds the greatest promise for transforming the clinical prognosis of ovarian carcinoma.

## Author Contributions

Yanru Long and Houhui Shi drafted the first version of this manuscript. Jiatian Ye critically revised the content of the draft. Xiaorong Qi supervised the completion of the manuscript and checked the final manuscript carefully. All authors have read and agreed to the published version of the manuscript.

## Funding

This work was supported by the Sichuan Province Tianfu Qingcheng Program Foundation, grant/award (No. KZ258).

## Ethics Statement

The authors have nothing to report.

## Conflicts of Interest

The authors declare no conflicts of interest.

## Data Availability

The authors have nothing to report.
